# Monitoring Virologic Responses to Antiretroviral Therapy in HIV-Infected Adults in Kenya: Evaluation of a Low-Cost Viral Load Assay

**DOI:** 10.1371/journal.pone.0006828

**Published:** 2009-08-28

**Authors:** Sumathi Sivapalasingam, Beatrice Wangechi, Fatuma Marshed, Maura Laverty, Shaffiq Essajee, Robert S. Holzman, Fred Valentine

**Affiliations:** 1 Division of Infectious Diseases, Department of Medicine, New York University School of Medicine, New York, New York, United States of America; 2 Bomu Medical Centre, Mombasa, Kenya; 3 Division of Infectious Diseases, Department of Pediatrics, New York University School of Medicine, New York, New York, United States of America; 4 Department of Environmental Medicine and Biostatistics, New York University School of Medicine, New York, New York, United States of America; 5 Department of Microbiology, New York University School of Medicine, New York, New York, United States of America; Sabin Vaccine Institute, United States of America

## Abstract

**Background:**

A key advantage of monitoring HIV viral load (VL) in persons receiving antiretroviral therapy (ART) is the ability to detect virologic failure before clinical deterioration or resistance occurs. Detection of virologic failure will help clarify the need for enhanced adherence counseling or a change to second- line therapy. Low-cost, locally performable alternates to expensive VL assays are needed where resources are limited.

**Methodology/Principal Findings:**

We monitored the response to 48-week ART in 100 treatment-naïve Kenyan adults using a low-cost VL measurement, the Cavidi reverse transcriptase (RT) assay and gold-standard assays, Roche RNA PCR and Bayer Versant HIV-1 RNA (bDNA) assays. In Altman-Bland plots, the mean difference in viral loads between the three assays was small (<0.5 log_10_ copies/mL). However, the limits of agreement between the methods exceeded the biologically relevant change of 0.5 log copies/ml. Therefore, the RT assay cannot be used interchangeably with the other assays to monitor individual patients. The RT assay was 100% sensitive in detecting viral loads of ≥400 copies/ml compared to gold-standard assays. After 24 weeks of treatment, viral load measured by the RT assay was undetectable in 95% of 65 patients with undetectable RNA PCR VL (<400 copies/ml), 90% of 67 patients with undetectable bDNA VL, and 96% of 57 patients with undetectable VL in both RNA PCR and bDNA assays. The negative predictive value of the RT assay was 100% compared to either assay; the positive predictive value was 86% compared to RNA PCR and 70% compared to bDNA.

**Conclusion:**

The RT assay compared well with gold standard assays. Our study highlights the importance of not interchanging viral load assays when monitoring an individual patient. Furthermore, the RT assay may be limited by low positive predictive values when used in populations with low prevalence of virologic failure.

## Introduction

An estimated 3 million HIV-infected patients in low- and middle-income countries were receiving antiretroviral therapy at the end of 2007 [1]. As the number of treated persons increases, so will the number failing treatment. World Health Organization (WHO) guidelines acknowledge the need to diagnose treatment failure using only clinical and/or CD4 T-cell criteria because high cost precludes the use of viral load assays in resource-limited settings (RLS) [2]. However, clinical and immunologic markers are poor predictors of virologic suppression [3], [4]. Furthermore, viral replication can increase months before immunologic or clinical deterioration, leading to resistance mutations that may limit future treatment options [5], [6]. Because, poor treatment adherence is the major cause of treatment failure of first-line HIV regimens [7], viral load measurements can help identify patients who might benefit from intensive adherence counseling and thus prevent, or at least postpone, a change to second-line therapy[8]. Despite these benefits, viral load monitoring remains inaccessible to the majority of patients on ART in RLS [9], [10].

One alternative to directly measuring the number of HIV RNA copies is to estimate it by measuring the activity of the HIV virus-encoded reverse transcriptase enzyme (RT) using the Cavidi ExaVir^®^ reverse transcriptase assay, a commercially available kit that is already used in some African countries. While gold-standard viral load assays such as Roche Amplicor HIV-1 Monitor (RNA PCR) and Bayer Versant HIV-1 RNA (bDNA) may cost ∼$80 per test, the RT assay costs approximately $21 dollars in Kenya. The equipment required for the RT assay is minimal and the assay can be conducted in a district- or provincial-level laboratory [11], [12]. An agreement signed in 2007 between Cavidi Tech and the Clinton Foundation to further reduce the cost of this assay to $12.50 per test will only increase its use (Fabio Baglioni, personal communication; Also see:http://www.cavidi.se/Templates/Cavidi/FileService.axd?id=38&v=1).

The RT assay has not been evaluated in a longitudinal field trial for monitoring patients on ART in RLS. Therefore, we monitored HIV-infected Kenyan adults initiating ART for 48 weeks using the RT assay, the RNA PCR, and the bDNA assays.

## Methods

### Study site

The study was conducted at the Bomu Medical Center (BMC), a non-governmental outpatient clinic located in an urban slum in Mombasa, Kenya. Since 2004, New York University School of Medicine has received funding from the Presidential Emergency Plan for AIDS Relief (PEPfAR) to provide free HIV care to patients attending BMC. ART treatment initiation criteria were CD4 count <200 cells/µL or WHO stage III or IV, irrespective of CD4 T-cell count [2]. The first line treatment regimen was the combination of stavudine (d4T), lamivudine (3TC), plus either nevirapine (NVP) or efavirenz (EFV). Zidovudine (AZT), didanosine (ddI) and lopinavir/ritonovir (Kaletra) were available for patients with drug toxicities or treatment failure.

One hundred consecutive ART-naïve patients were enrolled into the study if they were eligible to start ART and all provided written informed consent. The institutional review boards at Kenyatta National Hospital and at New York University School of Medicine approved this study.

Study visits occurred from 2004 through 2006, at baseline (before first ART dose) and weeks 4, 12, 24, 36 and 48. Each patient reported the number of doses of ART missed since the last visit. Perfect adherence was defined as reporting no missed doses since the last visit.

Viral load measurements were batch tested at the end of the study on plasma samples stored at −80°C. Viral load measurements were conducted on a single plasma sample that underwent a single thaw prior to testing. The Roche Amplicor HIV-1 Monitor Test, v1.5. (Roche Molecular Systems, Branchburg, NJ) procedure was performed, in blinded fashion, at the Kenya Medical Research Institute, according to the manufacturer's instructions [13]. Reported values for the Upper Limit of Quantitation (ULQ) and the Lower Limit of Quantitation (LLQ) were 750,000 (5.9 log_10_) copies/ml and 50 (1.7 log_10_) copies/mL, respectively. The VERSANT HIV-1 RNA 3.0 assay (Bayer Diagnostics, Tarrytown, NY) was performed at BMC according to the manufacturer's instructions by a technician trained by Bayer Diagnostics [14], [15]. The ULQ for this assay is 500,000 (5.7 log_10_) copies/ml, and the LLQ is 75 (1.9 log_10_) copies/mL. Due to a technical error in one run, nine samples for the bDNA assay were excluded from the analysis.

The Cavidi ExaVir^®^ Load Reverse Transcriptase Assay, version 2 assay was conducted in a blinded fashion according to the manufacturer's directions by Chem-Labs in Nairobi, the reference laboratory for Cavidi. Briefly, interfering enzymes in plasma were inactivated, virus was captured on a separation gel, drugs and other inhibitors washed away, and virions lysed to recover virion-associated RT enzyme. RT activity was measured by the enzymatic incorporation of bromdeoxyuridine (BrdU) into DNA captured on a poly-A coated enzyme immunoassay (EIA) plate, and the amount of incorporated BrdU was quantified by colorimetric detection. The results were calculated using the ExaVir^®^ Load Analyzer software and expressed in femtogram/ml (fg/ml) and equivalent RNA copies/ml (eq copies/ml). The ULQ for this assay varied in each run and ranged from 400,000 to 970,000 copies/mL, and the LLQ is 400 copies/ml.

Laboratory measurements included CD4 T-cell count, complete blood count, blood electrolytes, and liver function tests. CD4 cell counts were measured using Partec-GmBH (Partec, Münster, Germany) and FACSCount (Becton Dickinson Immunocytometry, Oxford, UK). No external quality assurance program was in place for either type of CD4 cell count instrumentation because this technology had been only recently introduced to treatment programs in Kenya. Twenty-seven patients did not have a CD4 cell count determination made after 24 weeks for the following reasons: 17 patients either died, were lost to follow-up or transferred to another institution and 10 patients either did not have CD4 counts ordered by the physician or there was insufficient blood volume. There was no statistically significant difference in terms of baseline CD4 cell count, baseline viral load, age, sex, or virologic failure between patients who did and did not have week 36 or 48 CD4 count.

### Statistical analysis

Although the lower limit of detection is different in the three viral load assays, we defined a “detectable viral load” as ≥400 copies/ml and “undetectable viral load as <400 copies/ml for all three assays. Sensitivity of the RT assay was the proportion of patients at baseline with detectable viral loads using the RT assay among those with detectable viral loads using RNA PCR or bDNA assays. Specificity of the RT assay was the proportion of patients after week 24 with undetectable RT activity among those with undetectable viral loads using RNA PCR or bDNA assays. The RT and RNA PCR or bDNA assays were compared using the Spearman correlation coefficient and the Altman-Bland plot [16]. The ability of the RT assay to differentiate between those with and without detectable RNA PCR viral load after week 24 was evaluated using the receiver operator characteristic (ROC) curve.

At 48 weeks, we defined the status of each patient as “in study,” “deceased,” “lost to follow-up” (missed 2 or more consecutive visits after week 24) or “transferred” to another facility. “Immunologic failure” was defined as a) a fall of CD4 count to pre-therapy level or b)>50% fall from the on-treatment peak, or c) a persistent CD4 level below 100 cells/ml [2]. “Virologic failure” was defined as either a) plasma RNA PCR viral load ≥400 copies/ml on two consecutive measurements after 24 weeks of therapy, following at least one undetectable viral load (<400 copies) after initiating ART or b) never achieving a RNA PCR viral load <400 copies/ml [2]. Values above the cut-off (≥400 copies/ml) were log_10_ transformed for analysis.

*Χ* ^2^ and Fisher's exact tests were used to compare categorical data and Student's *t* test was used to compare continuous variables. *P* values of <0.05 were considered to be statistically significant. Logistic regression was used to model variables associated with undetectable RNA PCR viral load. Patients who died, were lost to follow-up or transferred to another institution before the end of the study were not included in this analysis. Analyses were conducted using SPSS, version 11(SPSS Inc., Chicago, IL).

## Results

The median age of the 100 patients was 35 years (range, 5–68); 69 (69%) were women (Table 1) and the median baseline CD4 cell count was 147 cells/ml. Seventy-two (74%) of 97 patients who initiated ART, did so ≤1 year after HIV diagnosis. At week 48, 80 (80%) patients remained in care, 7 (7%) had died, 9 (9%) were lost to follow-up, and 4 (4%) transferred to another institution. Women were more likely to have remained in care (*P = *0.03). Otherwise, baseline characteristics (CD4 cell count, RNA PCR viral load, and age) did not differ between patients who remained in the study and those who did not. Among the seven patients who died, five died within 12 weeks after initiating therapy and two between 12 and 36 weeks.

**Table 1:** Baseline characteristics of study participants.

Baseline Characteristic^a^	Value^b^
Female sex—no. (%)	69 (69)
Age—no.(%)	
<10	2 (2)
11–19	0 (0)
20–29	32 (32)
30–39	43 (43)
40–49	28 (28)
>49	4 (4)
Ethnicity/Tribe –no.(%) (N = 96)	
Kamba	18 (19)
Luo	16(17)
Luhya	13(14)
Kikuyu	11(11)
Other (representing 18 different tribes)	38 (40)
Religion—no.(%) (N = 99)	
Christian	79(80)
Muslim	20(20)
Education—no.(%) (N = 94)	
None	9(10)
Primary school	41(44)
Secondary school	39(41)
College	5(5)
Marital status—no.(%) (N = 96)	
Married	48(50)
Divorced/separated	20(21)
Single	14(15)
Widowed	14(15)
Occupation—no.(%) (N = 97)	
Unemployed	41(42)
Small business	28(29)
Skilled manual labor	14(14)
Professional	8(8)
Unskilled manual labor	6(6)
Able to read –no.(%) (N = 96)	86(90)
Able to write –no.(%) (N = 96)	87(91)
Number of years between HIV diagnosis and initiating antiretroviral therapy—no.(%) (N = 97)	
≤1 year	72(74)
2 years	15(15)
3–5 years	8(8)
>5 years	2(2)
WHO Stage—no. (%)	
I	14(14)
II	23(23)
III	62(62)
IV	1(1)
Tuberculosis—no.(%)	9 (9)
Pneumonia—no.(%)	7(7)
Diarrhea—no (%)	25(25)
Received anti-malaria medication 1 month prior to initiating antiretroviral therapy—no. (%)	36 (36)
Pregnant—no.(%) N = 67	6(9)
CD4 T-cell count—per mm^3^	
Median	147
Interquartile range	61–197
HIV viral load (RNA PCR) copies/ml	
Median	204,500
Interquartile range	95,175–651,000
Hemoglobin—g/dl	
Median	10.6
Interquartile range	9.5–11.4
Initial antiretroviral-therapy regimen—no.(%) N = 93^c^	
Stavudine, lamivudine, efavirenz	53 (57)
Stavudine, lamivudine, nevirapine	33 (36)
Zidovudine,lamivudine,nevirapine	7 (8)

a N = 100 unless otherwise noted.

b Totals may not add to 100 because of rounding.

c Seven patients were lost to follow-up before treatment could be started.

### Comparison between the RT, RNA PCR and bDNA assays

**Figure 1:**
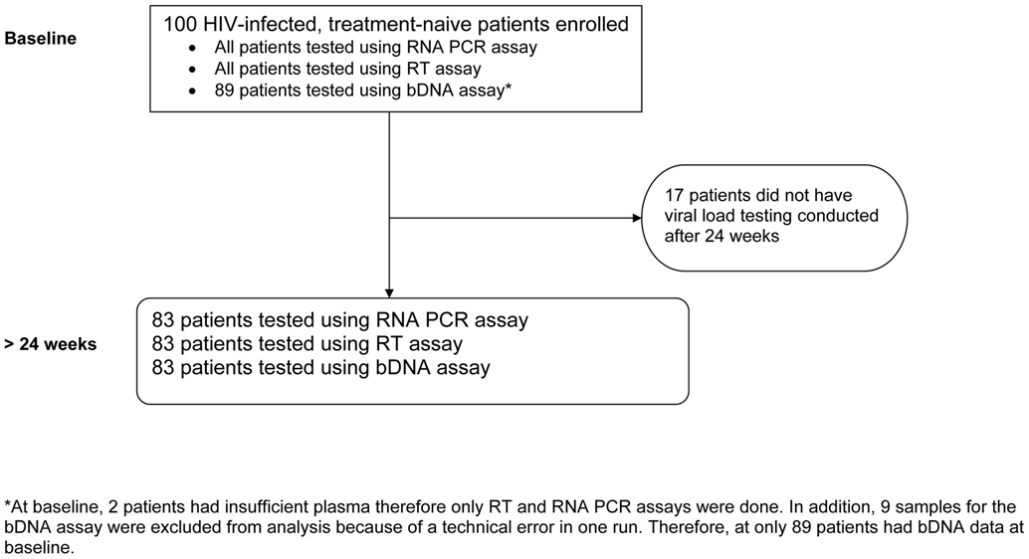
Flow chart of subjects with HIV viral loads conducted at baseline and follow-up after 24 weeks of antiretroviral therapy. (RT = Cavidi reverse transcriptase (RT) assay, Roche RNA PCR, and bDNA = branched DNA assay).

At baseline, 97 (97%) of 100 persons had detectable viral load by RNA PCR and 88 (99%) of 89 persons had detectable viral load by bDNA assay. Data from nine persons were excluded because of technical error in one bDNA assay run and two persons did not have sufficient sample volume to conduct all three assays at baseline (Figure 1). The sensitivity of the RT assay relative to the HIV RNA assay was 100% (97 of 97 patients with RNA PCR viral loads ≥400 copies/ml had detectable RT activity). The sensitivity of the RT assay relative to bDNA assay was 100% (88 of 88 patients with detectable viral loads measured by bDNA had detectable RT activity) (Table 2).

**Table 2:** Characteristics of the Cavidi^®^ reverse transcriptase assay for detecting HIV viral loads ≥400 copies/mL using RNA PCR or branched DNA assay (bDNA) assays in patients receiving antiretroviral therapy.

	Detecting HIV RNA PCR ≥400 copies/mL	Detecting bDNA ≥400 copies/mL
Sensitivity % (95% CI^a^) b	100 (96–100)	100 (96–100)
Specificity % (95% CI) b	95 (87–98)	90 (80–95)
Positive Predictive Value^c^ % (95% CI)	86 (65–95)	70 (49–84)
Negative Predictive Value^c^ % (95% CI)	100 (94–100)	100 (94–100)

a 95% CI, 95% Confidence Interval.

b Sensitivity of the RT assay was the proportion of patients at baseline with RT assays ≥400 eq copies/ml among those with viral loads ≥400 copies/ml using RNA PCR or bDNA assays. Specificity of the RT assay was the proportion of patients at week 36 or 48 of treatment (which ever was the last value) with undetectable RT activity among those with viral loads <400 copies/ml using RNA PCR or bDNA assays. bDNA data from nine patients were excluded due to technical errors in one run.

c Positive and negative predictive values were calculated using a 22% prevalence (18 of 83 patients with RNA PCR available after week 24) of a detectable RNA PCR viral load at week 36 or 48 of therapy; a sensitivity of 100% and specificity of 95% for the RNA PCR assay and a sensitivity of 100% and a specificity of 90% for the bDNA assay.

The specificity of the RT assay was 95% compared to the RNA PCR assay (62 of 65 patients with RNA PCR viral loads <400 copies/ml had undetectable RT activity) and 90% compared to the bDNA assay (60 of 67 patients with undetectable viral loads had undetectable RT activity). Among the 83 patients tested after 24 weeks of treatment, in eight, viral load was undetectable using the RT assay but detectable using either the RNA or bDNA assays. Five of the eight samples were RNA positive but bDNA negative, one was RNA negative but bDNA positive and two were negative in both RNA and bDNA assays. Therefore, the specificity of the RT assay was 96% compared to both RNA and bDNA assays (55 of 57 patients with both RNA PCR and bDNA viral loads <400 copies/ml had undetectable RT activity).

**Figure 2:**
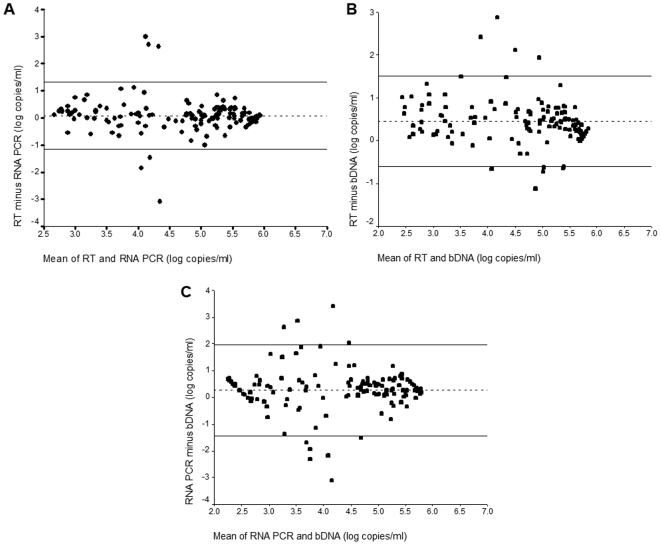
The measure of agreement between the RT assay and RNA PCR or bDNA assays is shown in the Altman-Bland plots. A. RT assay (equivalent log_10_ copies/ml) and RNA assay (log_10_ copies/ml); B. RT assay (equivalent log_10_ copies/ml) and bDNA assay (log_10_ copies/ml); and C. RNA assay (log_10_ copies/ml) and bDNA assay (log_10_ copies/ml). The dotted line represents the mean difference between values. Solid lines represent two standard deviations (2SD) (95% limits of agreement).

Correlations between RT and RNA PCR assays and RT and bDNA assays were statistically significant (Spearman correlation coefficients: 0.77, *P*<0.00001, and 0.66, *P*<0.00001, respectively). The measure of agreement between the RT assay and RNA PCR or bDNA assays is shown in the Altman-Bland plots in Figure 2. Because these plots visually assess the average difference in values between two assays over the usable range of the assays, only patients with detectable viral loads by the assays of interest were included. The mean difference between RT and RNA PCR assays was 0.09 log copies (95% CI: −0.008, 0.19) and the 95% limits of agreement (Mean+2SD, Mean - 2SD) were 1.32 log copies and −1.13 log copies. The mean difference between RT and bDNA assays was 0.45 log copies (95%CI: 0.35, 0.54) and the limits of agreement were 1.51 and −0.61 log copies. The mean difference between RNA and bDNA assays was 0.26 log copies (95%CI: 0.12, 0.40) and the limits of agreement were 1.96 and −1.44 log copies.

The area under the ROC curve was 0.89 (95% CI, 0.78–1.00) for identifying patients with virologic failure using the RT assay. The optimal inflection point was located at an RT viral load cut-off of ∼750 copies/ml (true positive rate: 90%; false positive rate: 8%).

### Virologic, immunologic and clinical response to ART

**Figure 3:**
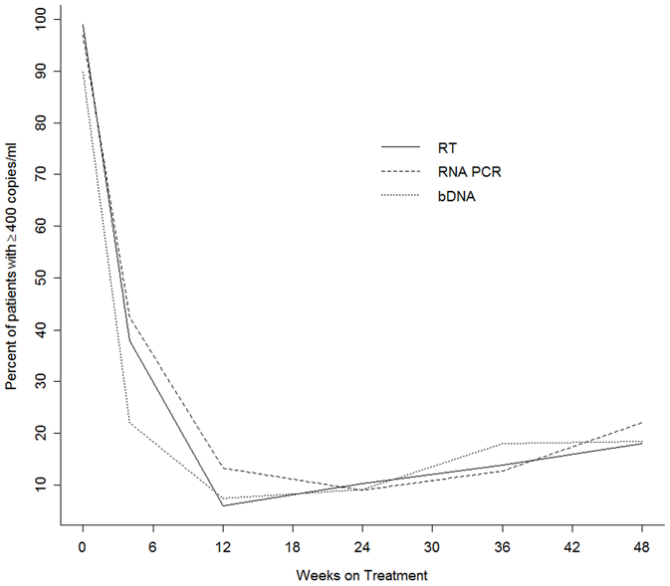
The proportion of patients with a detectable viral load (≥400 copies/ml) at study visits using three viral load assays (Cavidi reverse transcriptase (RT) assay, Roche RNA PCR, and branched DNA assay [bDNA]).

The median baseline RNA PCR viral load was 204,500 copies/ml (5.3 log_10_ copies). Six patients did not have a follow-up viral load after the baseline visit, and eleven did not have a follow-up viral load after week 24. At week 48 of treatment, 18% of patients had a single detectable viral load (≥400 copies/ml) using the bDNA and RT assays and 22% using the RNA PCR assay (Figure 3). When defining virologic failure as having two consecutive viral loads ≥400 copies/ml, among 83 patients who had viral load measurements conducted after 24 weeks of therapy, 10 (12%) had virologic failure; three never attained a viral load <400 copies/ml; three failed at 24 weeks and four at 36 weeks. The median RNA PCR viral load at the time of failure was 5,990 copies/ml (IQR: 1,540–287,250). In univariate analysis, patients with virologic failure were younger (32 years vs 35 years; *P* = 0.03) and were more likely to have imperfect adherence (OR 5.16, (95% CI: 1.20–22; *P* = 0.03) (Table 3).

**Table 3:** Characteristics associated with HIV RNA PCR virologic failure for antiretroviral naïve patients receiving antiretroviral therapy (ART), Mombasa, Kenya.^a^

Predictor variables	Univariate analysis	
	OR^b^ (95% CI)	*P*
Age	–	0.03^c^
Sex		
Male	Ref	
Female	3.95 (0.47–32)	0.20
Pregnant		
No	Ref	
Yes	3.78 (0.58–24.6)	0.16
WHO Stage		
Stage 1 or 2	Ref	
Stage 3 or 4	1.16 (0.30–4.4)	0.83
Highest level of education		
Primary school or none	Ref	
Secondary school or higher	2.71 (0.63–11.6)	0.18
Occupation		
Unemployed	Ref	
Employed	1.09 (0.28–4.1)	0.91
Adherence		
Perfect Adherence	Ref	
Imperfect Adherence	5.16 (1.2–22)	0.03
Baseline CD4 cell count		
<200 cells/ml	Ref	
≥200 cells/ml	1.78 (0.40–7.8)	0.45

a Excludes patients who died, were lost to follow up or transferred care to another institution.

b OR, Odds Ratio; CI, Confidence interval.

c By Student's *t* test.

**Figure 4:**
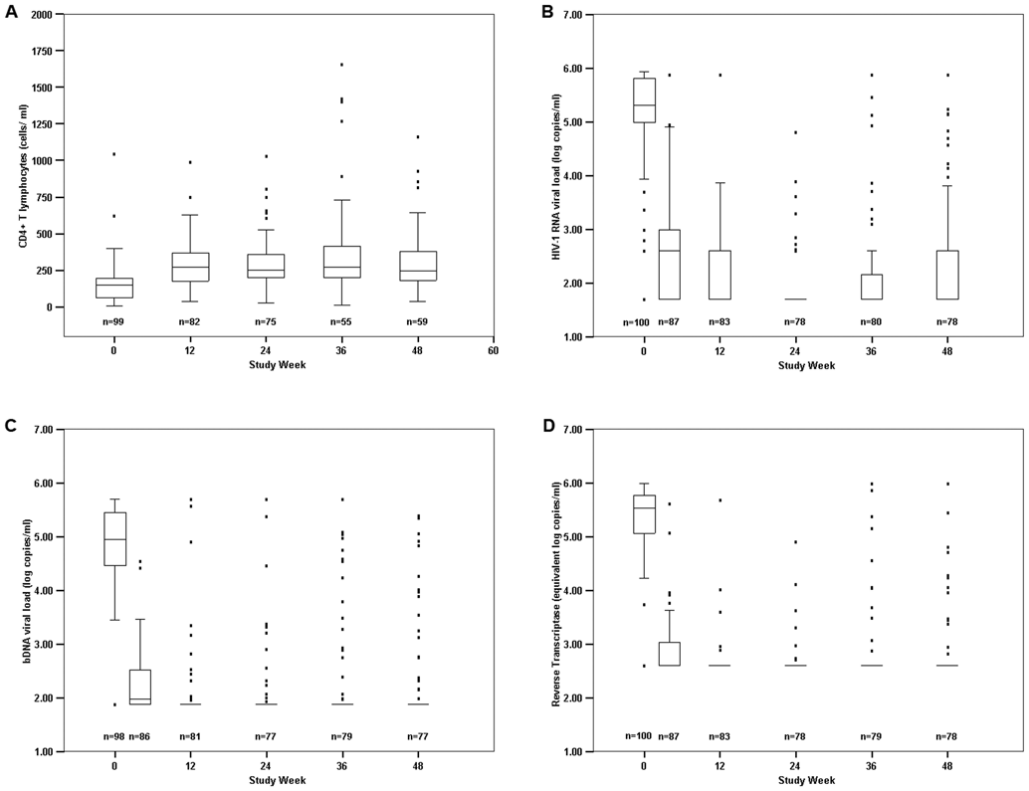
The distribution of CD4 cell count and HIV viral loads since initiation of antiretroviral therapy (study week 0). A. Distribution of CD4 cell count; B. HIV viral load by RNA PCR; C. HIV viral load by branched DNA (bDNA) assay; D. HIV viral load by Cavidi reverse transcriptase (RT) assay. Top and bottom of the boxes are 25th and 75th percentiles, respectively. Horizontal lines within the boxes indicate median values. Central vertical lines *(whiskers)* that extend from the boxes to the highest and lowest rates indicate the 95% and 5%, respectively. Dots indicate the individual values outside whiskers.

The median increase in CD4 T-cell count was 130 cell/mm^3^ (IQR, 34–247) (Figure 4). Seventy-three patients had CD4 cell count available at baseline and after week 24 of treatment. Eighteen patients had immunologic failure while on treatment; fourteen (78%) of these 18 patients had virologic suppression using RNA PCR.

## Discussion

We describe a longitudinal study of virologic treatment response to ART among antiretroviral-naïve Kenyan adults. Many received HIV testing late in their disease as demonstrated by the fact that 74% of those requiring ART were diagnosed within the prior year. The virologic failure rate, defined as a detectable RNA viral load on two consecutive measurements after 24 weeks of treatment, was 12%, with younger age and imperfect adherence being significant risk factors for failure. A wide range of virologic failure rates are reported from RLS, ranging from 4%–36%, in part because of the use of inconsistent definitions of virologic failure [17], [18], [19], [20], [21], [22], [23], [24], [25], [26]. We used a conservative definition of virologic failure, requiring two consecutive measurements of ≥400 copies/ml to avoid misclassifying intra-individual variations, laboratory errors, or transient elevations due to illnesses as true virologic failures.

The mean difference in viral loads between the RT assay, RNA PCR and bDNA assays as shown in the Altman-Bland plot was small (<0.5 log_10_ copies/ml) and did not vary systematically over the range of the assays. However, because the limits of agreement between the methods exceeded what is considered a biologically relevant change of 0.5 log copies/ml [27], we conclude that the RT assay cannot be used interchangeably with the RNA PCR or the bDNA assays. This caveat is also known to apply to the two gold standard viral load assays, and has led to the recommendation that a single assay be used to monitor treatment response in an individual patient in resource-rich countries [27]. Similar precautions will be even more relevant in RLS since HIV treatment programs may begin to offer viral load testing from different laboratories using different viral load technologies due to overwhelmed central laboratories.

Several studies have compared the ExaVir^®^ RT V.2 assay to gold standard viral load assays, however none was longitudinal [28], [29], [30], [31]. RT enzyme activity was detected in 95%–100% of samples having RNA PCR viral loads ≥50 copies/mL[31], ≥500 copies/mL [30], ≥1,000 copies/mL[28], and ≥2,000 copies/mL[29], and performed well for non-clade B HIV subtypes [29]. In our study, 95% of 65 patients with RNA PCR viral loads <400 copies/ml also had undetectable RT activity, while 97% of 65 patients with undetectable viral loads in both the RNA PCR and bDNA assays had undetectable viral load. Similarly, a study conducted in Botswana reported a specificity of 98% compared to RNA PCR [12]. In contrast, a recent study conducted in Kenya reported that only 12 (38%) of 31 samples with undetectable virus by RNA PCR had undetectable RT activity [31]. False-positive results given by the RT assay may indicate a lack of specificity of the test or laboratory error. An alternate possibility is that because the RT assay detects enzyme activity and therefore is not HIV sequence dependent, in contrast to gold-standard assays, false-positive results may actually indicate increased sensitivity of the RT assay in detecting recombinant or less common clades of HIV that are not detected by sequence-based assays. Since clade typing was not conducted in this study this remains a hypothesis. However, the potential for false-positive results using any of these assays warrants caution in using a single viral load measurement to trigger switch to second-line regimens.

Cost-effectiveness studies have shown that adding viral load monitoring is not cost-effective when second-line therapy is not available[32], but is cost-effective when second-line regimens are available [10], [33]. Furthermore, the cost-effectiveness ratio depends greatly on the viral load test price[10], [33] and rates of virologic failure[10]. In a recent South African study, in which the authors assumed that a single viral load of >1,000 copies/ml led to a switch to second-line therapy, reducing the per test cost of the viral load assay from $80 to $20 decreased the incremental cost-effectiveness ratio (ICER) from approximately $5,000 to $1,635 [10]. Interventions with an ICER between 1 and 3 times the gross domestic product (GDP) per capita are considered cost-effective, therefore extrapolating to Kenya ($1,600 GDP per capita in 2007), use of the RT assay (costing approximately $20 per test) would be considered a cost-effective intervention. The price of the RT assay is expected to decrease to $12.50 per test (personal communication, Fabio Baglioni, Cavidi AB) thereby further increasing the cost-effectiveness of this assay.

Many cost-effectiveness studies assume that a single high viral load would automatically trigger a switch to the more expensive second-line regimen[9], [10], [33]. This assumption likely drives up the cost of the viral load monitoring strategy unnecessarily. Programs have shown that viral load monitoring may, in fact, help to preserve first-line regimens through intensified adherence counseling [8], [34], [35]. Therefore, cost-effectiveness studies that include other consequences of monitoring viral loads in patients receiving ART, such as more intensive adherence counseling leading to better adherence and therefore fewer switches to second line regimen, may provide a more realistic cost of viral load monitoring in RLS.

Aside from cost, another advantage of the RT assay is that it does not require specialized laboratories or equipment and therefore can be conducted in a district or provincial laboratory, rather than a central laboratory [12]. Centralization of laboratory testing adds significant complexity and expense; increases opportunities for error in sample transport and transcription of patient identifiers and results; and increases turn-around times when testing volume is high. Although the three day turn-around time for the RT assay is often cited as a limitation of the assay[11], [12], in reality, sending samples to a central laboratory for viral load testing will likely result in longer turn-around times between collecting the sample and getting the test result because of various delays in shipping, sample processing and reporting.

There were several limitations to this study. Two different instruments in different laboratories were used to measure the CD4 cell count. This compromises the estimation of immunologic failure through changes in CD4 cell counts. However, previous studies substantiate our finding that using immunologic criteria to predict virologic failure is problematic [3], [4], [36]. External quality assurance programs were not available for the viral load assays. However all assays were performed using internal controls and by technicians trained by the manufacturer for the purpose of the study (bDNA) or in reference laboratories (RT assay and RNA PCR). Small sample size resulted in wide confidence intervals for the PPV therefore larger studies may be warranted before wide implementation of this assay is recommended. Finally, our study only included treatment-naïve patients. We cannot readily extrapolate the performance characteristics of the RT assay found in our study to treatment-experienced patients who may be infected with viruses with heavily mutated RT enzymes. Although preliminary data do not raise significant concerns [28], additional studies of the RT assay for monitoring treatment-experienced patients will be important.

Clearly defined strategies on the use of viral load monitoring technologies in RLS are not currently available and many questions remain, including whether viral load monitoring is even necessary [10], [37]. Access to a lower-cost assay that can be conducted in a district or provincial level laboratory is the first step to wider access to viral load monitoring. Overall, we found the RT assay compared well with gold standard assays in monitoring viral load responses to ART over 48 weeks making it a promising test for use in RLS.
